# Training-Induced Changes in Rapid Auditory Processing in Children With Specific Language Impairment: Electrophysiological Indicators

**DOI:** 10.3389/fnhum.2018.00310

**Published:** 2018-08-07

**Authors:** Anna Dacewicz, Aneta Szymaszek, Kamila Nowak, Elzbieta Szelag

**Affiliations:** ^1^Laboratory of Neuropsychology, Nencki Institute of Experimental Biology of Polish Academy of Sciences, Warsaw, Poland; ^2^Laboratory of Social Psychology, Department of Ergonomics, Central Institute for Labour Protection–National Research Institute, Warsaw, Poland

**Keywords:** specific language impairment (SLI), event related potentials (ERPs), temporal information processing, temporal windows, cognitive training

## Abstract

The brain’s ability to recognize acoustic changes occurring in rapid temporal succession is important for speech and successful language development. Children with specific language impairment (SLI) are characterized by deficient dynamics of temporal information processing (TIP) in the millisecond time range accompanied by disordered language development. Furthermore, previous studies have found that intervention based on amelioration of TIP resulted in improvement of both language and other cognitive functions. This study aimed to explain the changes associated with TIP training from the perspective of event-related potentials (ERPs). Thirty-six children aged 5–8 years (26 boys, 10 girls) diagnosed with SLI underwent two types of intense audio-visual computer intervention: experimental TIP training targeted at the millisecond time range (*n* = 18) or control non-TIP training (*n* = 18). Paired 50 ms tones of 1000 Hz and 1200 Hz were presented with inter-stimulus intervals (ISIs) of either 50 ms (Short ISI Condition) or 200 ms (Long ISI Condition). Auditory ERPs were measured in a passive oddball paradigm before and after each type of training. The mismatch negativity (MMN) paradigm was applied as an electrophysiological indicator of the brain’s ability to automatically detect violations of regularity in paired tones presented in rapid succession. Moreover, the P3a component was also analyzed. After 24 sessions of temporal training (in the experimental group) MMN amplitude enhancement was observed in both ISI conditions, reflecting increased efficiency in perceiving changes in rapid auditory sequences. In both experimental and control groups, P3a amplitude was enhanced in both ISIs. This may be due to the improvement of involuntary attention shifting to the auditory events involved in each training type. To conclude, temporal training, compared to non-temporal control training, improved the ability to detect changes in a rapid auditory stream in children with SLI.

## Introduction

### Temporal Dynamics of Cognitive Function

In recent years, temporal information processing (TIP) has been emphasized as the neural basis of several mental functions, such as learning, memory, attention, decision making, motor control and speech processing (Szelag et al., 2010). All these functions may be characterized by their specific temporal dynamics at different ranges of TIP. Two main ranges crucial to cognitive processes may be distinguished: millisecond and multisecond ranges (Pöppel, 1997, 2004, 2009; Buhusi et al., 2018). The temporal dynamics of these processes provide a framework for understanding the neural mechanisms underlying human mental activity, including a structure for speech and any perceptual or motor activity.

Furthermore, specific distortions of TIP have been found in various clinical populations (e.g., ADHD, autism spectrum disorder, depression, schizophrenia), co-existing with disordered psychological functioning; which may elucidate the consequences of deficient timing (e.g., Davalos et al., 2003; Teixeira et al., 2013; Vatakis and Allman, 2016). This line of research is important as it may foster our understanding of the “timing—behavior” relationship and potentially contribute to the remediation of psychological conditions. On this basis, one cannot claim that certain disorders are due to time distortions *per se*, but deviations from the “normal” template seem critical to everyday functioning and, hence, are a crucial factor for research and rehabilitation.

The number of experimental studies on the co-existence of “*deficient timing—deficient language*” in children and adults has grown rapidly. In this article, we concentrate on neurodevelopmental disorders, specifically on children with specific language impairment (SLI). Children suffering from SLI display problems with language acquisition (both comprehension and expression), however, their general cognitive functions and nonverbal intelligence remain in the normal range. Furthermore, these problems cannot be explained in terms of hearing deficits, neurological and speech mechanism abnormalities, or environmental factors. It is estimated that SLI affects approximately 7% of the 5-year-old population (Tomblin et al., 1997). In children with SLI, the coexistence of language difficulties and deficient TIP was first shown in an early article by Tallal and Piercy (1973) and subsequently confirmed in more recent studies (Grondin et al., 2007; Szelag et al., 2015). These findings raised the question of whether the improvement of TIP of nonverbal information may induce subsequent gains in language skills. Hence, the funding of training programs based on improvement of TIP to aid language competency has been given a high priority.

The widely used computer-based remediation Fast ForWord^®^ (FFW) has received a lot of attention in studies on improving language development in children. The program is based on the hypothesis that language impairments result from difficulties in rapid auditory processing (compare above). In the early studies of Tallal et al. (1996), as well as of Merzenich et al. (1996), it was found that after performing FFW exercises, there was a significant improvement in the language skills of children with expressive and/or receptive language difficulties and reading problems. However in some studies, the beneficial effects of FFW were comparable to those of other computer-based interventions (e.g., Cohen et al., 2005; Gillam et al., 2008; Given et al., 2008). Previous research on intervention-related effects indicated a transfer of improvement from the trained time domain to untrained behavioral domains, i.e., speech, auditory processing and other cognitive functions. Moreover, changes in brain function associated with auditory discrimination of both verbal (Lovio et al., 2012) and nonverbal stimuli presented in rapid sequences were reported (McArthur et al., 2010; Heim et al., 2013, 2016).

This research approach was applied in the Dr. Neuronowski^®^ computer program developed at our Institute (Szelag and Szymaszek, 2016). This program offers targeted training in millisecond TIP, sequencing abilities and duration judgment and has been developed on the basis of our many years of research (for an overview, see Szelag et al., 2009). Using this intervention program in our previous study on children with SLI, we found significant improvements in behavioral measures of timing, language, attention working memory and executive functions (Szelag et al., 2015).

### Time Perception Windows

As mentioned above, time perception can be considered on various time scales or processing units which are often called “temporal windows” (Pöppel, 1997). Experimental support for the existence of such time windows comes from a large number of different paradigms (Pöppel, 1997; Szelag et al., 2004). At this point, the close association between TIP and speech is well established and has been the topic of long discussions in modern neuroscience. This association may be rooted in the temporal dynamics of our verbal utterances which indicate the temporal constraints on different time ranges. These ranges correspond to tens of milliseconds (single phonemes), hundreds of milliseconds (syllables or prosodic elements), or a few seconds (phrases or sentences). Speech processing requires temporal decoding of the signal because the speech stream is a wave of rapidly changing complex sounds (millisecond processing level). Furthermore, it is necessary to parse the perceived signal into manageable chunks (multisecond level).

The concept of temporal characteristics of the speech signal was proposed in Poeppel’s (2003) Asymmetrical Sampling in Time hypothesis. This assumes that speech perception is associated with processing within two different temporal windows—shorter and longer windows—corresponding to two levels of processing: namely, phonological decoding and semantic parsing of the chunks of the incoming speech stream. Accordingly, the short window (~20–50 ms) is crucial for phoneme reception (e.g., rapid formant transitions, voice-onset-time), whereas the long window (~150–300 ms) contributes to syllable processing, intonation contour and prosody. Speech perception thus requires the integration of auditory information within these two ranges, which are the topic of the present study.

Existing data reveals that the length of such temporal integration windows (TIWs) of sensory information may be related to developmental fluctuations (Fox et al., 2010). For example, such TIWs are expected to be wider in children than in adults (Horváth et al., 2007; Fox et al., 2010) because the latter age-group can integrate information within shorter temporal segments than the former group. According to EEG studies, the length of the TIW in adults is around 200 ms, while in children of 5–8 years it is about 350 ms (Wang et al., 2005). It is also postulated that the length of such TIWs may change in subjects suffering from speech disorders.

### Mismatch Negativity (MMN) as an Indicator of “Genuine” Timing Efficiency

Different methods for measurement of millisecond timing efficiency have been developed (Szelag et al., 2004). One can distinguish here the classical behavioral methods, such as the detection of the temporal order of incoming events (Szymaszek et al., 2009). In these tasks, two consecutive stimuli are presented in rapid succession with a short gap in-between and the subject must report the order of occurrence (left-right, low-high, short-long, etc.). It is commonly believed that these tasks measure the effectiveness of sequencing ability and temporal ordering of incoming events on the basis of perceptual thresholds (auditory or visual) for order detection. Such threshold values are usually of tens of milliseconds in young healthy subjects (Wittmann and Fink, 2004; Szymaszek et al., 2009). However, the efficiency of TIP involves strong contributions from resources other than TIP, e.g., attention, working and short-term memory and moreover, inhibitory processes and decision making. Hence, behavioral measures of temporal acuity are heavily influenced by these cognitive functions.

On the other hand, the mismatch negativity (MMN) paradigm may be used as an electrophysiological indicator of timing efficiency in the processing of rapid auditory stimuli. The power of such event-related potentials (ERPs) lies in that they provide information about the sequencing, timing and in some cases, location of neural activity elicited by particular stimuli long before subjects produce an overt response. ERPs are increasingly used in developmental research because they are non-invasive and, in many cases, do not necessitate active subject participation, which is a huge advantage when dealing with children or patients.

MMN is a fronto-central negative potential elicited by any discriminable change in a sequence of auditory stimuli (Winkler, 2007; Näätänen et al., 2011). MMN can be induced by unattended stimuli, unlike behavioral methods which require attention and cooperation from a participant (Campbell and Davalos, 2015). Therefore, MMN can be a useful tool for measuring “genuine” timing deficits in subjects whose behavioral measures may be affected by attentional or other cognitive deficits.

MMN obtained in response to auditory stimuli has been proposed as a reliable objective method to measure sensory memory traces, as well as the effectiveness of rapid auditory discrimination processes (Davalos et al., 2003; Ervast et al., 2015). Therefore, MMN has been recognized as a sensitive indicator of auditory processing impairments in children with language difficulties (Davids et al., 2011), as well as in infants of parents with a history of language impairment (Benasich et al., 2002, 2006).

### Experimental Aim

This study aims to verify whether in children with SLI the application of the experimental temporal training compared to the control non-temporal training may result in enhanced electrophysiological responses in detection of changes in a rapid auditory stimuli stream. Another goal was to gain an understanding of the electrophysiological underpinnings of rapid nonverbal auditory processing in both the shorter and longer temporal windows. The application of the MMN paradigm allowed to concentrate on pure TIP, minimizing the influence of cognitive functions (attention, executive functions, etc.) involved in the behavioral timing measures.

## Materials and Methods

### Subjects

Participants were 36 children aged between 5 years and 8 years (26 boys, 10 girls) diagnosed with SLI according to ICD-10 (World Health Organization, 1992). They were recruited from either the Early Intervention Centre or the Children’s Memorial Health Institute in Warsaw. The core inclusion criterion was a language development disorder, defined as an overall standard score or score on at least two standard subtests below or equal to the 4th sten according to the Test for Assessment of Global Language Skills (TAGLS; Tarkowski, 2001), which constitutes the screening assessment for language development in Polish children. The recruited children scored below the mean score for their age. Moreover, all participants had a normal level of nonverbal intelligence (IQ of 85 or higher, measured with the Polish version of Raven’s Colored Progressive Matrices, CPM; Szustrowa and Jaworowska, 2003) and normal hearing level as verified by pure-tone audiometry screening at 500 Hz, 1000 Hz, 2000 Hz, 4000 Hz (using an AS208 audiometer), which covers the sound frequency spectrum used in this study. All children were monolingual Polish native speakers and were right-handed, based on the modified Edinburgh Handedness Questionnaire (Hill and Khanem, 2009). Participants had no neurological or psychiatric diagnosis, autism spectrum disorder, attention deficits, or socio-emotional disorders as determined by a parent questionnaire and clinician reports. Moreover, children did not attend any other speech or cognitive therapy while participating in this study.

### Ethical Approval

The study protocol was approved by the Bioethics Committee of the Medical University of Warsaw (permission no. KB/162/2010). Written informed consent was obtained from the parents of all children before the study. The children provided verbal approval before each session.

### Procedure

This was a blind study in which children were randomly assigned to two training groups using the RITA^®^ software (Pahlke et al., 2004) according to their age, gender, non-verbal IQ and level of language development: the Experimental Group (EG; *n* = 18; 13 boys, 5 girls) and the Control Group (CG; *n* = 18; 13 boys, 5 girls). These two groups did not differ significantly in terms of age, non-verbal intelligence, or level of language development. Descriptive data for EG and CG are shown in Table 1. EG underwent the computerized Dr. Neuronowski^®^ intervention program (Szelag and Szymaszek, 2016), focused on the enhancement of TIP in the millisecond time range. CG used a computer program which implemented classical speech therapy extended by 16 simple computer games. Thus, the intervention in EG and CG was matched in terms of the mental load, visual appearance, motivational aspects and training protocol with the exception of TIP exercises which were only included in the experimental training.

**Table 1:** Characteristics of the two groups of children with specific language impairment (SLI).

	Experimental group (EG)				Control group (CG)				Between-group differences	
	*n*	*M* (*SD*)	min	max	*n*	*M* (*SD*)	min	max	*t-value*^a^	*p-value*
Gender (boys/girls)	13/5	−	−	−	13/5	−	−	−	−	−
Age (years)	−	6.3 (1.0)	4.8	8.4	−	6.0 (0.8)	4.8	8.2	1.063	0.295
CPM (IQ)	−	111.5 (12.7)	92.6	135.9	−	114.6 (16.8)	88.4	141.5	−0.619	0.540
TAGLS (sten)	−	3.1 (1.7)	1	5	−	3.0 (1.5)	1	6	0.107	0.916

*CPM, Raven’s Colored Progressive Matrices; TAGLS, Test for Assessment of Global Language Skills. ^a^Two-tailed *t*-tests for independent samples*.

### Study Protocol

**Figure 1:**
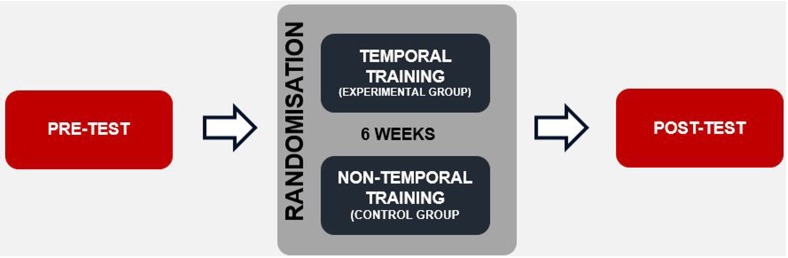
Schema of the experimental design.

Each participant underwent a *pre-test* electrophysiological assessment. Afterwards, the children underwent either experimental (EG) or control training sessions (CG; Figure 1). The duration of the whole intervention comprised 24 1-h sessions performed over 6 weeks (four sessions weekly). After completing the training program, a *post-test* was conducted using the same electrophysiological assessment as applied in the *pre-test*.

### Audiovisual Training Programs

Both trainings were computer-based intervention programs provided in several visually attractive exercises. To motivate and increase the children’s commitment to the training, exercises were conducted with the use of tablets because of some indications that this has a positive impact on young students (Dhir et al., 2013). Each child completed the training individually and each therapy session was guided by a trained consultant (speech-therapist, psychologist, special educator, or psychology student). The training took place in a separate room at the Nencki Institute or at the Early Intervention Centre.

In experimental and control training, both correct and incorrect answers were followed by appropriate visual and auditory feedbacks. The exercises in both interventions were conducted using a predefined specific agenda which allocated a comparable amount of time to training particular cognitive functions.

#### Experimental Training

The experimental intervention procedure was provided with the Dr. Neuronowski^®^ computer program developed at our Institute (Szelag and Szymaszek, 2016). The software consists of 46 games grouped into nine modules for improving particular cognitive functions (e.g., attention, non-verbal auditory processing, verbal short-term memory, executive functions, receptive language and phonemic hearing). In addition, the majority of these exercises involved millisecond TIP, sequencing abilities and duration judgment. The difficulty of particular exercises was adjusted individually on the basis of the actual level of a child’s performance.

The games mostly targeted the discrimination and identification of sounds, tones, syllables and words presented at a rapid rate, as well as recognizing the sequence or duration of two sounds, reproducing sequences of sounds or word strings and matching sounds and words.

#### Control Training

In contrast to the experimental training, none of the control intervention exercises involved TIP. The control training included three computer programs employing classical speech therapy and 16 simple computer games involving attention, short-term and working memory and executive functions.

The games employing speech therapy targeted the identification and discrimination of syllables and words presented at a regular rate of exposition as well as reproducing sequences of words or matching sounds and words. In the computer games, the tasks were to react as fast as possible to particular objects, to remember the pairs of sounds or pictures and to complete some logic puzzles.

### Electrophysiological Assessment

#### Procedure

**Figure 2:**
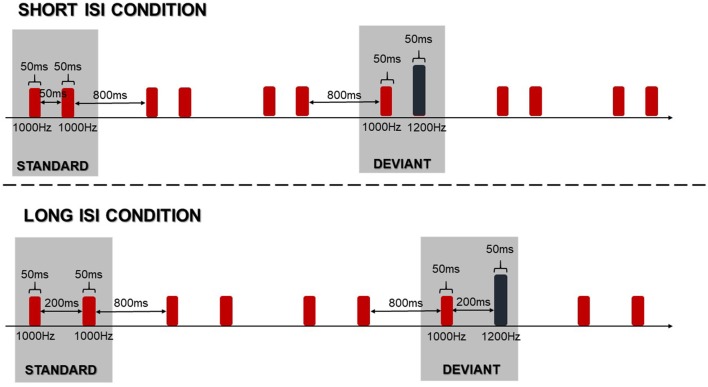
Schema of the passive oddball paradigm in two applied conditions.

In the electrophysiological assessment, children were exposed to paired sinusoidal tones in a passive oddball paradigm. This means that they were asked to ignore sounds while watching silent videos. The standard stimulus was a pair of identical tones at 1000 Hz, while the deviant stimulus was a pair of tones differing in pitch, at 1000 Hz and 1200 Hz. The duration of each pair of tones was 50 ms (with 5 ms rising and falling) with an intensity of 80 dB SPL, measured with a Brüel & Kjær sound level meter. The time between consecutive tone pairs was 800 ms. The schema of the passive oddball paradigm is presented in Figure 2.

Stimuli were delivered binaurally using Presentation Software version 14.9 (Neurobehavioral Systems Inc., Berkeley, CA, USA) via E·A·RTone^®^ 5A Insert Earphone headphones, inserted into the right and left ear canal. We implemented two conditions corresponding to the two inter-stimulus-intervals (ISIs) within paired-tones in standard and deviant stimuli. The ISIs were set at 50 or 200 ms creating the *Short ISI Condition* (Short ISI) and the *Long ISI Condition* (Long ISI), respectively (Figure 2).

In each condition, 600 standard and 150 deviant stimuli were presented (standard to deviant ratio was 75% to 25%) within six blocks (three blocks with Long ISI and three blocks with Short ISI) in two different orders randomized between subjects. In each block, 15 standards were presented at the beginning and the distribution of deviants was quasi-randomized: a minimum of 5 and maximum of 15 consecutive standards could be presented in a row. At the end of data collection, 300 deviant stimuli (150 for each condition) in four control blocks were also presented. This gave us an opportunity to use the same stimulus method (see below for description).

#### Data Acquisition

EEG data were recorded from 32 scalp electrodes (EasyCap, Germany) with Ag/AgCl active electrodes (ActiCAP, Brain Products) placed according to the 10-20 system of the BrainVision Recorder^©^ v.1.10 software (Brain Products, Germany). The electrodes’ contact impedances were kept below 10 kΩ. Data were referenced to the FCz electrode and a bandpass filter of 0.1–100 Hz was applied.

#### Data Analysis

Offline analysis was performed using BrainVision Analyzer^®^ v.2.0 software (Brain Products, Germany). First, the data were down-sampled to 256 Hz and re-referenced to TP9 and TP10 electrodes. Butterworth zero-phase filters were implemented with high-pass—1 Hz, low-pass—30 Hz (both 8 order), and notch filter—50 Hz. Next, artifacts were removed using Independent Component Analysis. The data were segmented into the epochs extending from 200 ms before to 1000 ms after the stimulus (standard or deviant) onset. After baseline correction (from −200 ms to 0 ms), trials exceeding ±120 μV were excluded from the analysis. Epochs were averaged for three stimulus types separately in the two conditions: standards, regular deviants and control deviants (deviants presented in control blocks).

**Figure 3:**
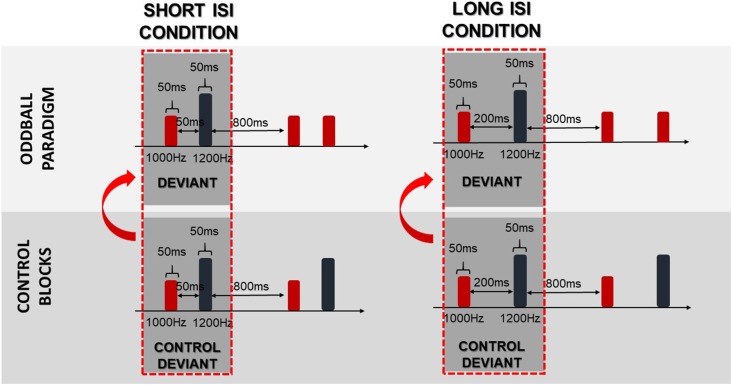
Schema of the same stimulus method. Arrows indicate that the difference waves were obtained by subtraction of the control deviants from the deviants.

To obtain difference waves dissociated from physical stimuli properties, the same stimulus method was used. In this method, the control deviants were subtracted from the deviants. The control deviants were physically identical to deviants presented in the oddball paradigm (regular), whereas control deviants were presented alone in a row in separate control blocks (Figure 3). As suggested in Jacobsen and Schröger (2003), a proper control block should control for both the physical properties of the stimuli and the adaptation effect. Nevertheless, in this study the numerous deviant repetitions in the control block did not fulfil the equal probability control block criteria, which might bias the reported effects. We refer to this issue in the “Discussion” section.

The main aim of this study was the analysis of MMN, which usually has frontocentral topography; hence the following analyses were performed only on FCz, Fz, F3, F4, FC1 and FC2 electrodes.

#### Waveform Analysis: Amplitude and Latency

The main analysis of data concerns ERPs identified on a difference wave, i.e., MMN and P3a, both before and after the interventions (experimental vs. control). Additionally, we performed explanatory analyses comparing N2 elicited by deviant and control deviant stimuli to the training-induced effects indicated by the MMN.

MMN and P3a amplitudes (or latencies) were obtained for each child. MMN was the most negative value in the time window between 195 ms and 395 ms after the first stimulus onset (i.e., 95–295 ms after the onset of deviation) for Short ISI or between 360 ms and 530 ms (110–280 ms after the onset of deviation) for Long ISI (e.g., Näätänen, 2000). The P3a component was the most positive value between 335–560 ms and 480–720 ms after the first stimulus onset for Short and Long ISI, respectively (e.g.,Escera and Corral, 2007). The maximum peak amplitudes for each participant were calculated in these time windows (Table 2).

**Table 2:** Time windows (in ms; from the onset of the first tone within a doublet) identified for particular event-related potentials (ERPs) analyzed in the study.

Condition	ERP	Deviant	Control deviant	Difference wave
Short ISI	N2	170–450	180–420	−
	MMN	−	−	195–395
	P3a	−	−	335–560
Long ISI	N2’	380–620	380–620	−
	MMN	−	−	360–530
	P3a	−	−	480–720

For deviants (presented in oddball) and control deviants (presented in separate blocks), P1-N2 and P1’-N2’ components were identified on averaged waveforms, which differed in Short and Long ISIs (Čeponienė et al., 2005). For Short ISI, only one electrophysiological response (P1 and N2) was elicited because of the integration of two tones within a pair. P1 was the most positive value, while N2 was the most negative one in the particular time windows shown in Table 2 for deviants and control deviants. For Long ISI, on the other hand, doubled electrophysiological responses were obtained, reflecting responses to two separate tones within a pair identified as P1-N2 and P1’-N2’ (e.g., Clunies-Ross et al., 2015). P1 and N2 were elicited by the first tone within the doublet, whereas, P1’ and N2’ by the second tone. In this study, we analyzed only N2 and N2’ components (in Short and Long ISI, respectively) because they reflected the electrophysiological response to the second tone within a deviant doublet (change onset). For this second tone, we got the MMN by subtracting the control deviant from the deviant. For each participant, the maximum peak amplitudes and latencies for N2 and N2’ were analyzed in the particular time windows (Table 2).

Finally, the amplitudes of particular ERPs (in μV) were determined as the maximum peak in the particular time windows with regard to the baseline. The latencies of MMN and P3a in both conditions were obtained by subtracting time intervals corresponding to the onset of stimulus change (i.e., 100 or 250 ms for Short and Long ISI, respectively).

## Results

### Statistical Analyses

To analyze differences in amplitudes (or latencies) of particular components in the two conditions, we conducted mixed-design analysis of variance (ANOVAs). Greenhouse-Geisser correction was applied when the sphericity assumption was violated. After ANOVAs, *post hoc* analysis was performed with the Bonferroni correction adjusted for multiple comparisons.

The data were analyzed in two stages.

In Stage 1, we examined the components identified on the difference wave, i.e., MMN and P3a for amplitudes (or latencies). In these ANOVAs, the within-subject factors were: *Session* (pre-test vs. post-test), *Condition* (Short ISI vs. Long ISI), whereas the between-subject factor was *Group* (EG vs. CG).

In Stage 2, to understand the intervention-related changes in MMN obtained in Stage 1, we examined the amplitudes (or latencies) of the N2 and N2’ waveforms observed for deviants and control deviants in the two groups, considering Short and Long ISI separately. The rationale was to clarify whether the training-related MMN amplitude changes resulted from increased or decreased amplitudes (or latencies) of N2 and N2’ elicited by deviants or control deviants. In the former case, the increased amplitudes would correspond to higher sensitivity of deviant detection in the oddball task, whereas in the latter case, the decreased amplitudes would result from repeated exposure. This may reflect reduced resources involved in the perception of a predictable auditory pattern. In Stage 2, the within-subject factors were: *Session* (pre-test vs. post-test), *Block* (deviants vs. control deviants) and *Group* (EG vs. CG) as a between-subject factor.

### Stage 1—Difference Wave

**Figure 4:**
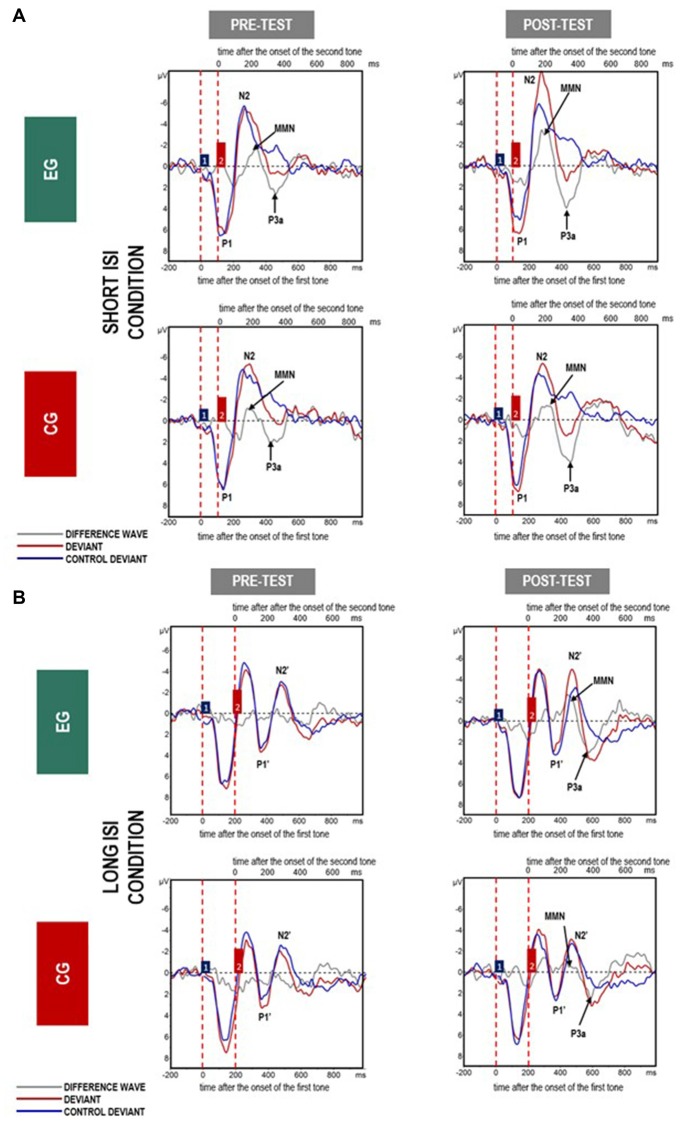
Grand average event-related potentials (ERPs) for all analyzed electrodes elicited in response to paired tones in **(A)** Short- and **(B)** Long inter-stimulus interval (ISI) for difference waves (gray line), deviants (red line) and control deviants (blue line) in experimental group (EG) and control group (CG) in *pre-* and *post-test*. The amplitudes of the waveforms are given in microvolts (μV) on vertical axes. Lower horizontal axes reflect the time distance after the change onset, i.e., the onset of the first tone within a doublet, whereas the upper axes reflect the time distance after the second tone onset. Vertical dashed red lines indicate the onset of the first and second tones within a doublet. Blue and red boxes (labeled by 1 and 2) reflect the first and second tones in the doublet.

**Figure 5:**
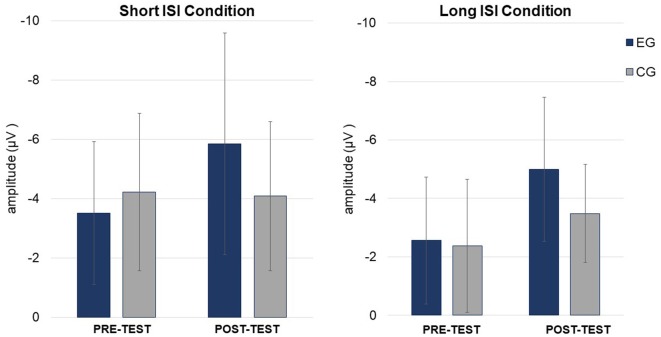
Mean mismatch negativity (MMN) amplitudes (in μV, with SD) in Short (left) and Long (right) ISI Conditions for EG and CG in *pre-* and *post-test*.

**Figure 6:**
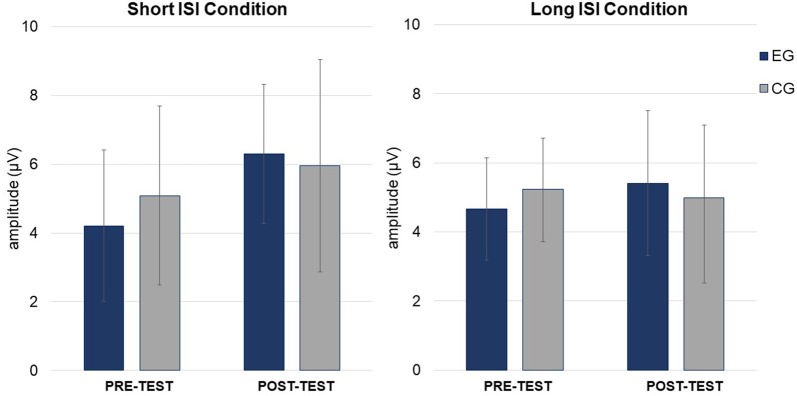
Mean P3a amplitudes (in μV, with SD) in Short (left) and Long (right) ISI Conditions for EG and CG in *pre-* and *post-test*.

Mean amplitudes for MMN and P3a are presented in Figures 4, 5, 6.

#### MMN

ANOVA conducted on amplitudes revealed significant main effects of *Condition* and *Session* modified by *Session* × *Group* interaction (see Table 3; Figure 5). The other factors and interactions were nonsignificant.

**Table 3:** Results of analyses of variances (ANOVAs) with repeated measures: *F*-values, *p*-values and effect sizes (*η*^2^) for the mismatch negativity (MMN) (top) and P3a (bottom) mean peak amplitudes (or latencies) including *Session* (pre-test vs. post-test) and *Condition* (Short ISI vs. Long ISI) as within-subject factors and *Group* (experimental group, EG vs. control group, CG) as a between-subject factor.

	MMN amplitude			MMN latency		
Effect	*F*	*p*	*η*^2^	*F*	*p*	*η*^2^
Session	13.635	0.001	0.286	1.790	0.190	0.050
Condition	7.600	0.009	0.183	3.934	0.055	0.104
Group	1.595	0.215	0.045	0.081	0.788	0.002
Session × Group	6.021	0.019	0.150	1.504	0.228	0.042
Session × Condition	0.855	0.362	0.146	0.154	0.697	0.005
Group × Condition	0.178	0.676	0.005	0.098	0.756	0.003
Session × Condition × Group	0.692	0.362	0.025	0.346	0.561	0.010
	**P3a amplitude**			**P3a latency**		
Session	4.376	0.044	0.114	14.686	0.001	0.302
Condition	0.932	0.341	0.027	5.467	0.025	0.139
Group	0.166	0.686	0.005	0.181	0.673	0.005
Session × Group	1.754	0.194	0.049	0.177	0.676	0.005
Session × Condition	3.743	0.061	0.099	1.647	0.208	0.046
Group × Condition	0.088	0.768	0.003	0.178	0.676	0.005
Session × Condition × Group	0.028	0.868	0.001	0.002	0.962	<0.001

*Significant effects are marked in gray background*.

The amplitudes were higher (more negative, *M* = −4.41; *SD* = 2.25) in the Short ISI than in the Long ISI (*M* = −3.36; *SD* = 1.73), irrespective of the *Session* and *Group*. Increased MMN in *post-test* was observed only in EG (*p* < 0.001). In CG, the difference between MMN amplitudes in *pre-* and *post-test* was nonsignificant (*p* = 0.387). The same pattern of results was observed for Short and Long ISIs. Although in *pre-test*, the two groups did not differ significantly in MMN amplitudes (*p* = 0.674), in *post-test* they were higher (more negative) in EG than in CG (*p* = 0.031). Mean and SD for MMN amplitudes are presented in Table 4.

**Table 4:** MMN (left) and P3a (right) mean peak amplitudes in μV (with SD) and latencies in ms in EG and CG in *pre-* and* post-test* and in Short and Long ISI Conditions.

		MMN amplitude		P3a amplitude	
Condition	Group	Pre-test	Post-test	Pre-test	Post-test
Short ISI	EG	−3.51 (2.41)	−5.85 (3.74)	4.21 (2.20)	6.29 (2.02)
	CG	−4.22 (2.65)	−4.08 (2.51)	5.08 (2.60)	5.96 (3.09)
	Total	−3.86 (2.52)	−4.96 (3.27)	4.64 (2.41)	6.12 (2.57)
Long ISI	EG	−2.56 (2.17)	−4.99 (2.47)	4.66 (1.48)	5.41 (2.10)
	CG	−2.38 (2.28)	−3.48 (1.68)	5.23 (1.52)	4.99 (2.47)
	Total	−2.48 (2.20)	−4.24 (2.21)	4.95 (1.50)	5.20 (2.27)
		**MMN latency**		**P3a latency**	
Short ISI	EG	212.87 (30.05)	193.28 (29.63)	365.30 (22.61)	340.35 (41.51)
	CG	202.63 (36.54)	202.90 (35.00)	351.74 (4.49)	333.46 (30.75)
	Total	207.75 (33.38)	198.09 (32.34)	358.52 (33.64)	336.91 (36.17)
Long ISI	EG	200.15 (28.70)	189.87 (34.68)	400.70 (102.36)	348.24 (53.18)
	CG	192.46 (30.18)	190.89 (37.53)	395.69 (104.95)	351.89 (73.91)
	Total	196.31 (29.28)	190.38 (35.62)	398.19 (102.20)	350.07 (63.48)

ANOVA conducted on latencies revealed a tendency toward significance (*p* = 0.055) for *Condition* with shorter MMN latencies in Long ISI (*M* = 193.34; *SD* = 25.69) than in Short ISI (*M* = 202.92; *SD* = 21.57). Mean (with SD) for MMN latencies are presented in Table 4 and Figure 5.

#### P3a

ANOVA conducted on amplitudes (Table 3) revealed a significant main effect of *Session*, with higher amplitudes in *post-* (*M* = 5.00; *SD* = 1.63) than *pre-test* (*M* = 4.37; *SD* = 1.27), independent of *Group* and *Condition* (*p* = 0.044).

In ANOVA on latencies (Table 3), main effects of *Session* and *Condition* were observed. Shorter P3a latencies were observed in *post-* (*M* = 343.49; *SD* = 41.12) than *pre-test* (*M* = 378.36; *SD* = 51.54; *p* = 0.001) and in Short ISI (*M* = 347.71; *SD* = 26.76) than in Long ISI (*M* = 374.13; *SD* = 66.48; *p* = 0.025) in both groups. Mean (with SD) for P3a amplitudes are presented in Table 4 and Figure 6.

In summary, increased amplitudes in *post-test* in comparison to *pre-test* were observed only in EG for MMN, but in both groups for P3a. Shorter P3a latencies were observed in *post-* compared to *pre-test* and in the Short ISI than in the Long ISI Condition.

### Stage 2—N2 and N2’

#### N2 (Short ISI Condition)

In ANOVA (*Group* × *Session* × *Block)* on amplitudes, a main effect of *Block* modified by the *Session* × *Block* interaction was observed (see Table 5). N2 amplitudes were higher in *post-* (*M* = −8.91; *SD* = 4.17) than in *pre-test* (*M* = −7.62; *SD* = 3.39) but only for deviants (*p* = 0.006). For control deviants, this difference was nonsignificant (*p* = 0.531). Moreover, only in *post-test* were the amplitudes for deviants (*M* = −8.91; *SD* = 4.17) higher than for control deviants (*M* = −7.15; *SD* = 2.59; *p* < 0.001). In *pre-test*, the amplitudes for deviants and control deviants did not differ significantly (*p* = 0.676).

**Table 5:** Results of ANOVAs with repeated measures: *F*-values, *p*-values and effect sizes (*η*^2^) for the N2 (left) and N2’ (right) mean peak amplitudes (or latencies) including *Session* (pre-test vs. post-test) and *Block* (Deviants vs. Control deviants) as within-subject factors and* Group* (EG vs. CG) as a between-subject factor.

	N2 amplitude			N2’ amplitude		
Effect	*F*	*p*	*η*^2^	*F*	*p*	*η*^2^
Session	1.858	0.182	0.052	8.859	0.005	0.207
Block	6.678	0.014	0.164	4.084	0.051	0.107
Group	3.936	0.055	0.104	1.279	0.266	0.036
Session × Group	12.465	0.001	0.268	4.203	0.048	0.110
Session × Block	11.400	0.002	0.251	3.696	0.063	0.098
Group × Block	0.196	0.660	0.006	4.812	0.035	0.124
Session × Block × Group	3.332	0.077	0.089	0.136	0.714	0.004
	**N2 latency**			**N2’ latency**		
Session	0.010	0.922	<0.001	1.167	0.288	0.033
Block	1.263	0.269	0.036	3.141	0.085	0.085
Group	1.487	0.231	0.042	2.557	0.119	0.070
Session × Group	2.502	0.123	0.069	0.035	0.852	0.001
Session × Block	2.060	0.160	0.057	1.122	0.297	0.032
Group × Block	0.157	0.694	0.005	0.056	0.814	0.002
Session × Block × Group	0.215	0.646	0.006	0.352	0.557	0.010

*Significant effects are marked in gray background*.

The *Session* × *Group* interaction resulted from higher amplitudes in *post-* than *pre-test* in EG (*p* = 0.001, see Table 5). In CG the difference between sessions was nonsignificant (*p* = 0.135). Moreover, in *post-test* in EG, amplitudes were higher than in CG (*p* = 0.003). *Pre-test* between-groups differences were nonsignificant (*p* = 0.644). Mean (with SD) for N2 amplitudes are presented in Table 6.

**Table 6:** N2 (left) and N2’ (right) mean peak amplitudes in μV (with SD) and latencies in ms in EG and CG, in *pre-test* and* post-test* and in Short and Long inter-stimulus interval (ISI) conditions.

		Short ISI condition		Long ISI condition	
		N2 amplitude		N2’ amplitude	
Block	Group	Pre	Post	Pre	Post
Deviants	EG	−7.71 (4.17)	−10.75 (4.78)	−4.71 (2.71)	−6.60 (3.90)
	CG	−7.51 (2.51)	−7.07 (2.40)	−3.83 (1.80)	−4.62 (1.75)
	Total	−7.62 (3.39)	−8.91 (4.17)	−4.27 (2.31)	−5.61 (3.15)
Control deviants	EG	−7.78 (3.26)	−8.40 (2.74)	−3.46 (1.26)	−4.66 (1.98)
	CG	−7.07 (2.90)	−5.90 (1.74)	−4.40 (1.81)	−4.18 (1.76)
	Total	−7.42 (3.06)	−7.15 (2.59)	−3.93 (1.61)	−4.42 (1.86)
		**N2 latency**		**N2’ latency**	
Deviants	EG	287.24 (44.38)	292.44 (38.10)	523.69 (81.90)	504.82 (87.81)
	CG	316.82 (52.74)	293.44 (34.48)	508.11 (65.58)	482.41 (36.38)
	Total	302.03 (50.32)	292.94 (35.82)	515.90 (73.55)	493.61 (67.21)
Control deviants	EG	275.78 (39.20)	294.22 (56.58)	553.11 (89.88)	542.98 (109.09)
	CG	293.76 (59.15)	296.26 (47.04)	518.43 (77.34)	523.74 (70.56)
	Total	284.77 (50.29)	295.24 (51.29)	535.77 (84.49)	533.36 (91.07)

In ANOVA on N2 latencies, all effects were nonsignificant (see Table 6).

#### N2’ (Long ISI Condition)

In ANOVA (*Group* × *Session* × *Block)* on amplitudes, the main effect of *Session* was significant (*p* = 0.005), whereas* Block* tended towards significance (*p* = 0.051). Two interactions, *Session* × *Group* and *Group* × *Block*, were observed (see Table 5).

The *Session* × *Group* interaction resulted from higher amplitudes in *post-* than in *pre-test* in EG (*p* = 0.001). In CG, these differences were nonsignificant (*p* = 0.517). Mean (with SD) for N2’ amplitudes are presented in Table 6.

Although the groups did not differ significantly in amplitudes for deviants (*p* = 0.083) or control deviants (*p* = 0.617), the *Group* × *Block* interaction reflected higher amplitudes in EG in response to deviants (*p* = 0.005) than to control deviants. These differences in CG were nonsignificant (*p* = 0.904). Mean (with SD) for N2’ amplitudes are presented in Table 6.

No significant effects in ANOVA on N2’ latencies were observed (see Table 5).

In summary, enhanced N2 amplitudes *post-test* compared to *pre-test* were observed for deviants (presented in oddball), indicating increased deviant detection sensitivity. These differences were nonsignificant for control deviants. In EG, enhanced N2 and N2’ amplitudes for both deviants and control deviants were found in *post-test*. These amplitudes were higher in response to deviants than to control deviants.

## Discussion

This study showed important training-related influences *post-test* as compared to *pre-test*: (1) in EG in *post-test* increased MMN was accompanied by enhanced N2 and N2’ amplitudes for deviants (in oddball); (2) in both groups *post-test*, higher P3a amplitudes and shorter latencies were observed. Additionally, in Short ISI, generally higher MMN amplitudes were observed irrespective of training effect.

The applied MMN paradigm allowed the study of the “genuine” timing, minimizing the influence of other cognitive processes which are highly involved in any behavioral task. It is known that MMN is generated automatically, even without overt attention being paid to the presented stimuli and it reflects the brain’s pre-attentive ability to detect any violation of the regularity of auditory stimulation (Näätänen and Picton, 1987; Winkler, 2007). Due to its low cognitive demands (such as attention, decision making, executive function, etc.) it is considered a reliable and objective measure in children. In our study, the use of MMN allowed the verification of whether temporal training caused changes in electrophysiological responses as compared to the control non-temporal training.

Below, we discuss the observed relationships in terms of training-induced effects and general rapid auditory processing.

### Training-Related Changes in Rapid Auditory Processing

#### MMN

After training, increased MMN amplitudes in both conditions were obtained only in EG (Figure 4). This result is congruent with data from other studies indicating increased MMN amplitudes after temporal-auditory interventions (Kujala et al., 2001; Heim et al., 2016). For example, Heim et al. (2016) reported normalized ERP amplitudes and latencies of MMN in children with language-learning impairment after the administration of FFW (see “Introduction” section). Nevertheless, they also found enhanced MMN *post-test* in healthy controls, probably due to task re-exposure. As in our study, increased MMN was only found in EG, excluding any effect of repeated measurement but suggesting, rather, that the changes were training-related. Moreover, Kujala et al. (2001) found increased MMN amplitude in response to paired tones following 14 sessions of auditory discrimination training in children with dyslexia. They reported a correlation between increased MMN amplitude and improved reading skills. In contrast, in our study we did not find any statistically significant correlations between enhanced MMN and improvement in behavioral measures reported in our previous article (Szelag et al., 2015). This lack of correlation in young children may be caused by the high variability of behavioral indicators which are highly cognitively demanding.

In our study, the training-related improvements indexed by MMN amplitudes in EG were *post-test* by both enhanced N2 and N2’ amplitudes for the deviants, but not for the control deviants (see “Results” section, Stage 2). This may suggest increased temporal acuity after temporal training and better detection of the regular deviants. These relationships were not observed in CG.

At this point, some conclusions can be drawn about the potential impact of the control block design on the obtained results. As mentioned before, our control block allows the stimuli’s physical properties to be controlled while ignoring the adaptation effect. One should agree that the reported MMN amplitudes could potentially be artificially elevated because of habituation due to repetition of the control deviants. As significant differences between sessions for control deviant amplitudes were not found, one may expect that the adaptation effect modified both the pre- and post-test measurements in a similar way. Therefore, we expect that our control block design will only have weakly influenced the training-related changes, which were the main focus of our study. However, we would recommend that future studies be more careful in designing the control block, as suggested by, e.g., Jacobsen and Schröger (2003).

The effects of our temporal training may be important for improving the rapid auditory processing of nonverbal information. There are convincing indications that such processing is the neural basis of language and speech (see “Introduction” section). The training-induced improvements in TIP may result in a transfer of the improvement from the time domain to the language domain in which timing is incorporated. Thus, improving timing in nonverbal information may facilitate speech functions. This seems to be crucial for future clinical applications of temporal training procedures in speech therapy in subjects with language disorders (aphasia or SLI; Szelag et al., 2014; 2015; Szymaszek et al., 2017).

According to previous studies, other auditory trainings for children with language disorders (e.g., FFW, Earobics, phonological interventions) may induce changes on different levels of auditory processing. For example, Pihko et al. (2007), in a MEG study, observed a stronger response to syllables, as reflected in the enhancement of both the P1 component and MMN, corresponding to speech improvement indexed by behavioral measurements in bilingual SLI children. Training-related changes were also observed in ABRs (auditory brainstem responses) in children with SLI or CAPD (central auditory processing disorders; Filippini et al., 2012) indicating that auditory processing may be enhanced at very early stages (subcortical stages).

The transfer between improved auditory processing and language processing after auditory interventions (musical trainings) may be associated with enhanced sensitivity to acoustic features creating a base for speech improvement (Besson et al., 2011).

#### P3a

Some training-related changes were also observed in both groups in the P3a component, which reflects involuntary attention shifting (Escera and Corral, 2007). After both interventions, enhanced amplitudes and shortened P3a latencies were found, irrespective of condition. Our results are similar to those obtained by Lovio et al. (2012) who reported P3a enhancement after both the experimental (phonological awareness) and control (math games) interventions, whereas MMN increment were observed only following the experimental intervention. This pattern of training-related improvements is similar to that found in the present study, i.e., enhanced MMN in EG and increased P3a in both groups.

This training-related P3a increment in our study in both groups may be caused by some features common to the two interventions. These are: (1) similar attentional engagement; (2) comparable mental load; and (3) involvement of auditory discrimination (compare the “Materials and Methods” section). Thus, after both kinds of intense training, involuntary shifting of attention to the presented auditory stimuli might be enhanced, independent of the condition. Moreover, some researchers have claimed that language improvement induced by audiovisual trainings in children with language disorders may be moderated by attentional processes (Cohen et al., 2005; McArthur et al., 2010). Stevens et al. (2008) observed enhanced ERPs associated with selective attention in children with SLI after FFW administration resulted in receptive language improvement. Some support for this observation also comes from children with dyslexia showing diminished P300 amplitudes as well as better behavioral performance in a lexical decision task after audiovisual training (Jucla et al., 2010). The interpretation is that after such training, attentional resources are more efficiently allocated during word recognition.

Regardless of the training approach taken, there is room to create new intervention tools for children with language learning impairments.

### Two Temporal Mechanisms of Auditory Perception in SLI

Looking at the waveforms displayed in Figure 4 for deviants (red lines) and control deviants (blue lines), we can assume that two mechanisms of auditory processing were active depending on the duration of the ISI separating the two stimuli presented in rapid succession. This may be reflected in two different patterns of neural responses. For Short ISI (paired tones separated by an ISI of 50 ms), one electrophysiological response was elicited and indicated by a complex P1-N2 response which is dominant in children (Ervast et al., 2015). This reflects a situation in which the response to the second tone within a pair began before the termination of the response to the first tone, which is reported in studies using paired stimuli separated by a relatively short ISI (Figure 4). Evidence from previous literature has suggested the existence of a 200 ms long TWI following the onset of the first sound, in which a unitary sound representation is formed (Näätänen and Winkler, 1999;Horváth et al., 2007). In contrast, for longer ISIs (above 200 ms) two distinct neural responses were obtained: P1-N2 followed by P1’-N2’ for the first and second tone within a pair, respectively. The electrophysiological response to the second tone seemed to appear after termination of the response to the first tone. Similarly, two separated MMN potentials were elicited when the stimulus onset asynchrony exceeded the duration of the TWI (Long ISI Condition; Figure 4).

Such unitary sound representation has been reported in previous studies. For example, Wang et al. (2005) and Fox et al. (2010) indicated the integration of two paired tones presented with relatively short ISIs. Fox et al. (2010) obtained two distinct neural responses in children aged 7–9 when paired tones were separated by a 200 ms ISI. For ISIs of 25, 50 or 100 ms, only one neural response was elicited. Furthermore, Wang et al. (2005), using the *double deviant* method in children aged 5–8, reported two separate MMN responses elicited by two kinds of deviant presented in a row differing either in pitch or intensity, but only when the separating ISI was 250 ms. At shorter ISIs (i.e., 50, 100, or 200 ms) they found only one MMN, indicating an integrated neural response to two different kinds of deviants. In summary, the above research on temporal integration focused on an approximately 200 ms border interval known as the TIW. This research has clearly indicated the elicitation of one neural response when such an interval is short or near the TIW limit, whereas, two distinct neural responses are observed when the time window following the onset of a sound exceeds the TIW.

The important observation is that the children with SLI studied here presented the same pattern of responses as their normally developing peers (reported in previous studies), i.e., one integrated MMN response at shorter ISIs and two separate responses at longer ISIs. We may speculate that despite the TIP deficits in children with SLI evidenced in behavioral methods, the neural basis of TIP seems preserved. As reported in our previous studies (Szelag et al., 2015), children with SLI displayed deteriorated perception of the temporal order of two sounds presented in rapid succession, i.e., they displayed higher temporal order thresholds (values of approx. 200 ms) than their typically developing peers (approx. 100 ms). Our results are promising as they show that the neural mechanism of TIP evidenced in the electrophysiological method reported here is intact. One might expect that this preserved neural base may constitute the framework for the efficacy of TIP training, resulting in improved language skills.

### Short vs. Long ISI Detection Reflected in MMN

Higher MMN was observed in Short ISI as compared to Long ISI (Figure 4). As higher amplitudes are usually observed in easier tasks than in more difficult ones (e.g., Näätänen, 2000), one may expect that for children with SLI, processing in Short ISI was easier than in Long ISI. This was confirmed by greater MMN amplitudes in the former case, irrespective of Session and Group (see “Results” section, Stage 1, Figure 4). It seems that processing within the TIW (Short ISI) fosters easier detection of violation of regularities. Conversely, if the stimulus-onset-asynchrony exceeds the TIW, smaller MMN (or even no MMN) occurred. The Long ISI Condition required a longer memory trace which made the detection of violation more difficult.

According to Näätänen (2000), MMN occurs when the memory trace of the standard is still active during deviant presentation, thus, MMN may depend on sensory memory persistence. The overall duration of the paired-stimulus pattern in Short ISI (150 ms) was twice as short as that in Long ISI (300 ms), resulting in greater sensory memory load in the latter case, creating more a difficult perceptual situation. On the other hand, according to Winkler (2007), increased MMN amplitude in response to the paired-tone patterns may indicate the improvement of temporal grouping of incoming sounds, which seems crucial in the process of auditory object formation.

We emphasized the advantage of the MMN paradigm in testing TIP in children as it does not require the participant to actively perform the task or even to attend to the stimuli. In this respect, this paradigm has a large advantage over behavioral studies. It is worth mentioning that results of electrophysiological and behavioral measurements examining the same phenomena cannot be compared directly. Considering the behavioral data previously reported in children with SLI (Szelag et al., 2015), an opposite relation of detection may emerge—i.e., shorter ISIs may make the temporal ordering more difficult than long ISIs. Szelag et al. (2015) indicated that thresholds of temporal order for two sounds required an ISI of approx. 200 ms, whereas at shorter ISIs it was reported at a chance level. This suggests that longer ISIs created an easier perceptual situation than shorter ones. Such divergence between the electrophysiological results presented here and the behavioral data published previously may be due to the different mechanisms implemented in the sequencing of the auditory stimuli presented with the various ISIs, i.e., perceptual auditory streaming or identification of separate sounds.

## Conclusion

In summary, using ERP we have demonstrated that temporal training may enhance the detection of changes in rapid auditory streams in children with SLI. Improved temporal resolution in two temporal windows was measured in terms of increased MMN. Our study also indicated that intense cognitive training (both temporal and non-temporal) in children with SLI may ameliorate involuntary attention shifting as reflected by increased P3a amplitudes.

Results indicate that, not only in normal children (studied by, e.g., Fox et al., 2010) but also in children with SLI, auditory perception of sequences of rapidly changing stimuli depends on the duration of the ISI separating the presented stimuli. At a relatively short ISI (50 ms), one electrophysiological response was elicited, whereas at a long ISI (200 ms) two separate electrophysiological responses occurred.

## Author Contributions

AD: subject recruitment, data acquisition, conducting therapy sessions, analysis and interpretation of data, contribution to manuscript writing. AS: data acquisition, conducting therapy sessions, analysis and interpretation of data, manuscript writing. KN: conducting therapy sessions, analysis and interpretation of data. ES: conceptualization and study design, analysis and interpretation of data, manuscript writing. All authors: final approval.

## Conflict of Interest Statement

ES and AS are the creators of the Dr. Neuronowski^®^ software package, realized as part of a project at the Nencki Institute with funding from the National Centre for Research and Development in Poland. The rights to the software lie with the Nencki Institute, which has an agreement with Harpo Ltd., the company commercializing this software. ES and AS are not the owners of this technology nor do they have a direct financial arrangement with Harpo Ltd. The authors state that this does not affect the scientific validity of the results. The remaining authors declare that the research was conducted in the absence of any commercial or financial relationships that could be construed as a potential conflict of interest.
